# Primary health care contribution to improve health outcomes in Bogota-Colombia: a longitudinal ecological analysis

**DOI:** 10.1186/1471-2296-13-84

**Published:** 2012-08-16

**Authors:** Paola A Mosquera, Jinneth Hernández, Román Vega, Jorge Martínez, Ronald Labonte, David Sanders, Miguel San Sebastián

**Affiliations:** 1 Department of Public Health and Clinical Medicine, Epidemiology and Global Health, Umeå University, 901 87 Umeå, Sweden; 2 Postgraduate courses in Health Administration and Social Security, Pontificia Universidad Javeriana, 40 623 Bogota, Colombia; 3 Institute of Population Health, University of Ottawa, K1N 6 N5 Ontario, Canada; 4 School of Public Health, University of the Western Cape, P Bag X17, Bellville 7535, South Africa

**Keywords:** Primary health care, Health outcomes, Population Health, Outcomes Assessment, Multivariate analysis, Bogota

## Abstract

**Background:**

Colombia has a highly segmented and fragmented national health system that contributes to inequitable health outcomes. In 2004 the district government of Bogota initiated a Primary Health Care (PHC) strategy to improve health care access and population health status. This study aims to analyse the contribution of the PHC strategy to the improvement of health outcomes controlling for socioeconomic variables.

**Methods:**

A longitudinal ecological analysis using data from secondary sources was carried out. The analysis used data from 2003 and 2007 (one year before and 3 years after the PHC implementation). A Primary Health Care Index (PHCI) of coverage intensity was constructed. According to the PHCI, localities were classified into two groups: high and low coverage. A multivariate analysis using a Poisson regression model for each year separately and a Panel Poisson regression model to assess changes between the groups over the years was developed. Dependent variables were infant mortality rate, under-5 mortality rate, infant mortality rate due to acute diarrheal disease and pneumonia, prevalence of acute malnutrition, vaccination coverage for diphtheria, pertussis, tetanus (DPT) and prevalence of exclusive breastfeeding. The independent variable was the PHCI. Control variables were sewerage coverage, health system insurance coverage and quality of life index.

**Results:**

The high PHCI localities as compared with the low PHCI localities showed significant risk reductions of under-5 mortality (13.8%) and infant mortality due to pneumonia (37.5%) between 2003 and 2007. The probability of being vaccinated for DPT also showed a significant increase of 4.9%. The risk of infant mortality and of acute malnutrition in children under-5 years was lesser in the high coverage group than in the low one; however relative changes were not statistically significant.

**Conclusions:**

Despite the adverse contextual conditions and the limitations imposed by the Colombian health system itself, Bogota’s initiative of a PHC strategy has successfully contributed to the improvement of some health outcomes.

## Background

Primary Health Care (PHC) is considered to be an effective strategy to improve accessibility and utilisation of services, address the social determinants of health inequities and improve health outcomes by the implementation of comprehensive interventions through sectoral and intersectoral collaboration, empowerment of individuals, social mobilisation and community participation [1-4].

In Colombia, a simplified and limited conception of PHC was introduced in the official health plans in the 1980s within the old National Health System (NHS). In 1993, Act 100 reformed the previous NHS into the current General System of Social Security in Health (GSSSH), undermining the principles of PHC articulated in the 1978 Alma Ata Declaration [5] by creating a health system based on insurance markets with different public-private provider combinations.

With this reform, public health programmes were the responsibility of the local governmental health authorities and individual health services the responsibility of insurance companies [6-8]. Public health activities are included in a benefits package known as the Collective Intervention Plan (CIP—Plan de intervenciones colectivas in Spanish) which complements individual health services included in the Compulsory Health Plan [9]. This division of functions has generated a highly segmented and fragmented national health system.

Despite Colombia having adopted a health system based on neoliberal market principles [9], Bogota in 2004, as part of a center-left government (elected for first time in the city) decided to reinstate the PHC principles as one of their strategies to improve the quality of life and the level of population health, and to reduce health inequities. This initiative was possible due to the local level decentralization of the health system in Colombia. Thus, the PHC strategy emerged as a purely local effort (from the Mayor of the city, the District Health Secretariat, the public health care network and the community) without receiving neither political nor technical support by the national health system and within a context of constraints imposed by the insurance market rationality. The reforms began to be implemented through the programme “Home Health” (“Salud a su Casa” in Spanish). The programme operates in the network of first level public hospitals operating under the authority of the Bogota District Health Secretariat (DHS). The essential elements of the strategy included a rights-based approach rooted in community participation, empowerment of social groups and intersectoral work [10,11].

The core operative elements of the PHC “Home Health” programme [6,10-12] were: the organisation of multidisciplinary basic health care teams, with each team assigned to 1200 families prioritizing the most vulnerable people (strata 1 and 2)^a^; the articulation and integration of individual and collective actions to improve personal health through the implementation of actions in the settings of daily life including families, neighbourhoods, schools, kindergarten and workplaces; the implementation of an intersectoral response focused on solving community needs; and the promotion of social participation as a right, thereby creating opportunities for community organisation and mobilisation.

Several studies have found that health systems based on PHC principles ensure better access and higher quality of health services, a high level of satisfaction among users, better health levels in the population, an increase in social participation and lower costs [4,13-19]. In Latin America the association between the improvement of health conditions and the implementation of PHC has been reported in several studies, which have identified positive impacts on infant mortality rate (IMR), post-neonatal mortality and under-5 mortality rate [15-19].

Reports from the “Home Health” programme and an assessment analysis of the PHC implementation carried out by the DHS in Bogota have shown that an increase in the number of basic health care teams has contributed to a rise in the number of activities of preventive programmes, vaccination coverage [20,21] and social participation processes [11,12]. Additionally, a preliminary study examining at the possible association between PHC implementation and improvement of health outcomes showed that IMR, post-neonatal and under-5 mortality due to pneumonia had decreased in areas with a high “Home Health” programme coverage compared with areas with low coverage. This research recommended the inclusion of socio-economic variables in order to confirm the findings [19].

The current study aimed to analyse the contribution of the PHC strategy, through the increase of coverage of the “Home Health” programme, to the improvement of health outcomes in Bogota.

## Methods

### Study design

A longitudinal ecological design was carried out using data from secondary sources to evaluate associations and possible effects of the implementation of PHC through the “Health Home” programme on selected health outcomes, controlling for socioeconomic variables.

### Study Settings

Bogota, Colombia's capital has 7.035.155 inhabitants and is divided geographically and administratively into 20 localities. The city government is headed by the Mayor and localities also have a local mayor. According to the social stratification, 51.2% of the population is classified in strata 1 and 2. The PHC strategy have been included in the District health policy of the last two periods of government; however this strategy has been facing unstable conditions since its beginning due to the lack of a constant source of resources. The efforts of some localities to enhance PHC expansion have depended mainly on political and administrative will.

### Units of analysis and variables

Sixteen of 20 localities were included in this study as units of analysis. Four localities were excluded because three did not have a population in strata 1 and 2, and the other lacked socioeconomic information necessary for the analysis.

Dependent variables were health outcomes identified in the literature as sensitive to PHC implementation [22,23]: infant mortality rate, under-5 mortality rate, infant mortality rate resulting from acute diarrheal disease (ADD) and pneumonia, prevalence of acute malnutrition in children under 5 years of age, vaccination coverage for diphtheria, pertussis, tetanus (DPT) in children under 1 year of age and prevalence of exclusive breastfeeding among infants under 6 months. The data were collected from the National Vital Statistics System, the Feeding and Nutrition epidemiological surveillance systems and the Rapid Immunisation Coverage Monitoring Registry at DHS.

Independent variables were those related to PHC coverage intensity: coverage of characterisation^b^ to the “Home Health” programme and health personnel ratio (physicians, nurses and health promoters) per population target of the programme. The health personnel ratio was calculated by full-time equivalents for each type of personnel and the estimation of the population target of the programme (strata 1 and 2) by using retrospective projections with the information from the District Secretariat for Planning in 2002, 2009 and 2010, complemented with data from the 2005 population census from the National Administrative Statistics Department.

As confounder variables, socioeconomic indicators available in the District Quality of Life Survey (DQLS) of 2003 and 2007 were selected: the quality of life index (QLI)^c^, the population below the poverty line, the household dependency ratio, the proportion of consumption reduced due to lack of money, the sewerage coverage and the insurance coverage to the contributory and subsidised regimes^d^.

Data from dependent and independent variables were used with authorization of the public health department at the District Health Secretariat. Data from socioeconomic variables were taken from public available sources.

### Data analysis

Initially, a Primary Health Care Index (PHCI) was constructed, which combined the variables of PHC coverage intensity (coverage of characterisation to the “Home Health” programme and health personnel ratio per population) using principal component analysis. This index summarises the behaviour of variables earlier mentioned in each year. The PHCI was standardised giving values from 0 to 100.

According to the PHCI, localities were classified into two groups: the first composed of those localities where the PHCI increased during the first years, but then declined or became stagnant (low coverage/group 1); and the second composed of localities that showed a consistent increase of PHCI over time with an expansion of the population included in the programme and in the number of basic health care teams (high coverage/group 2).

Subsequently, a multivariate analysis was conducted to assess associations between health outcomes and PHCI in each group while controlling for the socioeconomic variables. The models included, in addition to health outcomes and the PHCI groups, sewerage coverage, health system insurance coverage and quality of life index (QLI). Population below the poverty line, household dependency ratio and the proportion of consumption reduced due to lack of money were not included because of high collinearity with the QLI. The analysis was performed using data from 2003 (the year previous to the “Home Health” programme implementation) and 2007 (third year after the implementation), because of the lack of socioeconomic information for other years. First, a *Poisson regression model* for each year was developed separately and rate ratios (RR) adjusted for the socioeconomic variables calculated. Afterwards, in order to assess changes in the health outcomes between the groups over the years, a *panel Poisson regression model* was used. In this model, each year was included as a panel and the difference among the groups compared. A random-effects model was applied after the alternative approach, the fixed-effects model, was rejected because the Hausman test was not statistically significant. The analysis was carried out using STATA 11.

### Ethics approval

This study was approved by the Ethics Committee of the department of postgraduate courses in Health Administration and Social Security at Javeriana University.

## Results

**Figure 1:**
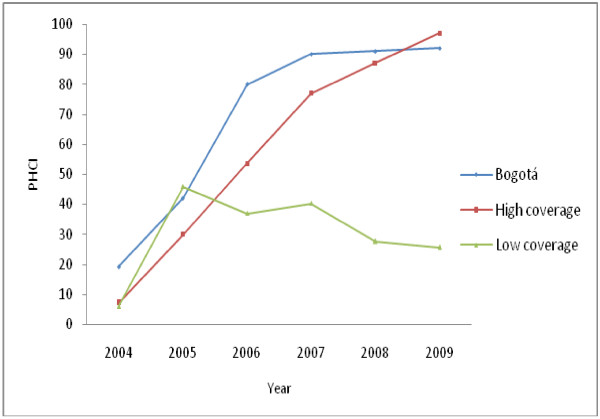
Trends of PHCI in Bogota and localities grouped into high and low coverage (2004–2009).

Figure 1 presents the evolution of the PHCI in Bogotá, which showed a notable initial increase between 2004 and 2007, followed by a period of slower growth between 2007 and 2009. As mentioned above, with the results of PHCI on a disaggregated level, each locality was classified in one of the two groups (high or low coverage).

Table 1 presents socioeconomic and health characteristics for 2003 and 2007. As regards the socio-economic conditions, a statistically significant increase of the QLI was observed, a decrease in enrollment in the contributory regimen and an increase in enrollment in the subsidised regimen as well as a slight increase in sewerage coverage. The “Home Health” programme began its expansion in 2004 and had covered a third of the population in strata 1 and 2 by 2007. Overall there was an improvement in the health outcomes. IMR, under-5 mortality rate, and infant mortality rate by ADD showed a slight decrease while infant mortality rate by pneumonia decreased 75% and the prevalence of acute malnutrition 25% between 2003 and 2007. Prevalence of exclusive breastfeeding showed a slight increase while the vaccination coverage for DPT increased 25% by 2007.

**Table 1:** Mean values of the variables analyzed for 16 Bogota’s localities (2003–2007)

**Variables**	**Mean 2003**	**Mean 2007**	**Change 2007–2003**	
QLI	89,42	90,25	0,83*	
Insurance coverage to the contributory regimen (%)	80,87	73,25	−7,62*	
Insurance coverage to the subsidized regimen (%)	19,13	24,37	−5,24*	
Households with access to sewerage (%)	99,14	99,6	0,46	
Coverage of Home Health program (%)	0	33,14	33,14*	
Under-5 mortality rate (Per 10000 children under 5 years)	32,81	31,14	−1,67	
Infant mortality rate (per 1000 live births)	15,42	14,24	−1,18	
Infant mortality rate by ADD (per 1000 live births)	0,26	0,105	−0,16	
Infant mortality rate by pneumonia (per 1000 live births)	9,74	0,71	−9,03*	
Acute malnutrition in children under −5 years (% of children under 5)	6,4	5,2	−1,20*	
Prevalence of exclusive breastfeeding (% of infants under 6 months)	70,8	73,9	3,10	
Vaccination coverage for DPT (% of children under 1)	81,5	91,9	10,40*	

*p<0,05 from paired *t*-test.

Table 2 presents the results of the *Poisson regression model* for each year. In the same table, the results of the p*anel Poisson regression models* that analyse the relative changes between 2007 and 2003 are shown.

**Table 2:** Results of the multivariate analysis (Poisson regression) 2003 and 2007 and the relative changes between 2007/2003 for the selected health outcomes

**Variables**	**2003**			**2007**			**Relative change 2007/2003**		
	**RR‡**	**[95% CI]**		**RR‡**	**[95% CI]**		**RR‡**	**[95% CI]**	
**Under-5 mortality rate**									
Low coverage	1			1			1		
High coverage	0.915	0.773	1.084	0.879	0.765	1.011	0.862*	0.780	0.953
**Infant Mortality rate**									
Low coverage	1			1			1		
High coverage	1.067	0.892	1.277	0.951	0.819	1.104	0.968	0.870	1.078
**Infant Mortality rate by ADD**									
Low coverage	1			1			1		
High coverage	1.367	0.272	6.866	2.052	0.290	14.50	1.078	0.353	3.294
**Infant Mortality rate by pneumonia**									
Low coverage	1			1			1		
High coverage	0.957	0.474	1.932	0.629	0.314	1.262	0.625*	0.400	0.976
**Acute malnutrition in children under −5 years**									
Low coverage	1			1			1		
High coverage	0.866*	0.795	0.944	0.723*	0.662	0.790	0.945	0.894	1.000
**Prevalence of exclusive breastfeeding**									
Low coverage	1			1			1		
High coverage	0.954	0.903	1.007	0.961	0.921	1.003	0.968	0.935	1.004
**Vaccination coverage for DPT**									
Low coverage	1			1			1		
High coverage	0.879*	0.859	0.900	1.05*	1.030	1.072	1.049*	1.034	1.063

* Difference between low and high coverage statistically significant (p < 0.005).

‡ After adjusting for QLI, insurance coverage and sewerage coverage.

In 2007, high coverage localities had a lower risk of acute malnutrition and a higher probability of being vaccinated than low PHCI coverage localities. Results showed statistically significant reductions of the risk of under-5 mortality (13.8%) and infant mortality by pneumonia (37.5%) in the high-coverage group between 2003 and 2007. Despite the risk of infant mortality and acute malnutrition in children under-5 years also showing a decline in the high coverage group, the relative changes were not significant.

The probability of being vaccinated for DPT significantly increased (4.9%) between 2003 and 2007 for the high coverage group and although the prevalence of exclusive breastfeeding also increased, it was not statistically significant.

## Discussion

The analyses presented here suggest that PHC coverage through the “Home Health” programme is associated with improvements of some health outcomes over time in Bogota. The results have confirmed through a multivariate analysis the findings of the descriptive assessment conducted in 2008 [19], which found that health outcomes were better in those localities with high coverage of the “Home Health” programme.

Our results are also consistent with evidence from other contexts [15-18,24-30] where an increase in PHC coverage has been related to improvements in child health.

In the Latin American region, this association has been widely evidenced. For example, an analysis of 22 countries identified a general decrease in IMR and under-5 mortality rates related to increasing PHC coverage between 1990 and 1998 [25]. Similarly, a study from Costa Rica in 2004 reported that for every 5 years of the reform that introduced PHC to its health system, the IMR was reduced by 13% [18].

Moreover, a comparative analysis between regions with different levels of PHC development in Brazil showed a greater reduction in IMR in those regions where the “Family Health” programme had been well implemented [15,16]. During the period 1990–2002, Brazil reported that an increase of 10% in the coverage of the “Family Health” programme was associated with a reduction of 4.6% in IMR, while controlling for other socioeconomic variables [15]. Subsequently, in the period 1999–2004 this finding was confirmed reporting an attributable reduction of 13% in the IMR, 16% in the post-neonatal mortality and 44% in infant mortality rate due to ADD [16]. Similarly, a Bolivian study found lower IMR (35.1%) and under-5 mortality rates (74.3%) in PHC intervention areas when compared with non-intervention areas; and a decrease in the mortality rates by more than half (52%) in the intervention areas 5 years after the PHC implementation [17].

In other low and middle income countries in Africa and Asia reductions in the IMR have also been described ranging from 20% to 65% as a result of the strengthening of PHC interventions [12]. A research study in five East African countries concluded that three quarters of the risk to IMR could be decreased with the enhancement of PHC, including interventions such as antenatal care, immunisation and the provision of potable drinking water [26].

Studies from South Africa have reported decreases between 10% and 32% of acute malnutrition in children under 5 years of age [13,27], and comparisons made in Bolivia suggested a lower risk of mortality due to acute malnutrition in areas where PHC has been better developed [17]. As regards the increase in vaccination coverage, different studies in developing countries have shown significant correlations between increases in PHC coverage and availability of immunisation [13,16,17,28]. An association between PHC coverage and increases in the prevalence of exclusive breastfeeding has also been supported by different reports, suggesting that access to preventive programmes for maternal and child care is enhanced by PHC interventions. In this respect, programmes based on PHC could strengthen important components of preventive health activities and may be easier and faster than other approaches in achieving a higher prevalence of exclusive breastfeeding [29,30].

The results of our study differ from those reported in the Brazilian studies regarding infant mortality rates due to ADD and pneumonia. Brazil reported statistically significant decreases in infant and post-neonatal mortality rates due to ADD and non-significant reductions in mortality from acute respiratory infections [15,16]. In our case, it was the opposite.

Contextual factors emerge as possible explanations for the Bogota findings. The significant reduction in mortality as a result of pneumonia could be due to the introduction of “Acute Respiratory Disease rooms” (“Salas ERA” in Spanish) as part of the PHC strategy [19]. However, this intervention could not be considered in this analysis because of the lack of available information. The reasons for a higher risk of mortality due to ADD in high coverage localities, although not statistically significant, are not clear. It might be that the government has developed more intensively in low PHCI localities certain social interventions related to diarrheal diseases such as improving water and sanitation, increase of sewerage coverage and implementation of programmes that provide food subsidies.

Moreover, it is important to note that the magnitude of the effect in this study was lower than that observed in other contexts. This could be because in the present analysis only data for the third year after the implementation of PHC were included and a longer period of time would have been required to demonstrate a greater effect. Another possible explanation suggested in one of the first assessments of the Brazilian programme [15] is that IMR and under-5 mortality rates had already experienced a significant decline years before the PHC implementation as function of a wide range of social interventions that could affect health outcomes, and that the scale of future declines would be less sensitive to the interventions associated with PHC reforms.

### Study limitations

The limited scope of the ecological design does not provide conclusive evidence of causality. Likewise, the unavailability of information on a disaggregated level lower than localities (e.g. micro-territories, families or individuals) does not permit us to determine with certainty whether the reductions or increases in health outcomes occurred in the targeted population.

In addition, the lack of information about population size in each strata before 2009 led us to carry out retro-projections that are only an estimate of the population size. This could under- or overestimate the variables that make up the PHCI and therefore could affect the measurement of the coverage intensity and the classification of groups.

Another important limitation is the periodicity and availability of socioeconomic information, which is collected only every four years; this could have affected our estimations of the relationship between the PHC coverage and the improvement of health conditions.

Finally, the complexity and influence of many social determinants on the health outcomes studied ideally requires a multi-level analysis that takes into account latent unmeasured variables that could be confounding the apparent relationship between PHC and health outcomes. Further research should include representative data on individual “Home Health” users and non-users and additional variables that allow a disaggregation of the evolution of macro and micro social indicators. Such research could improve efforts to disentangle which factors of the PHC reforms are contributing to improvements in health outcomes.

## Conclusions

The overall findings of this study showed that increases of PHC coverage through the “Health Home” program were related to improvements in the health status of Bogota’s population. A high coverage of the program was significantly related to lower under-5 mortality rate, infant mortality by pneumonia and higher vaccination coverage for DPT.

Our results also provide further evidence to support the hypothesis that health conditions improve when the coverage of PHC increases; and that some interventions offered by the PHC strategy, such as health education, access to health services, referral and inclusion in nutrition programmes and the promotion of social participation, help to improve population health status.

This results point out that, despite the adverse contextual conditions and the limitations imposed by the Colombian health system itself, the promotion of a PHC strategy has successfully contributed to the improvement of health outcomes in the population. Given these results, it is necessary to establish a stable fund of resources to ensure the sustainability and equitable distribution of the PHC strategy allowing the expansion of the “Home Health” programme coverage.

## Endnotes

^a^Strata classify socio-economic groups from 1 to 6, 1 being the lowest and 6 the highest. This classification determines the taxes and prices of home public services as well as access to health service among others.

^b^Characterisation is the first activity carried out by the basic health care teams in the “Home Health” programme in order to include the families of strata 1 and 2. This consists of the application of a survey which identifies the socio-economic and health conditions of the family.

^c^QLI combined 12 variables of access to physical assets organised into four categories: 1) Education and human capital: education of the household head, average education of members from 12 years or more; young people aged 12–18 years who attend secondary school or university; children aged 5–11 years who attend primary school; 2) Housing quality: material of walls and floors; 3) Access and quality of services: access to health care, water supply and sanitation, kitchen equipment, refuse collection; and 4) Household size and composition: number of children under 6 years of age and number of people per room.

^d^In the Colombian health system, individuals are usually enrolled to one of two different regimes: contributory, for those with employment where the person pays for the service, and the subsidised, for those without capacity to pay where the person receives a subsidy according to the poverty status.

## Competing interests

The authors declare that they have no competing interests.

## Authors’ contributions

PM, JH, RV and JM conceived the study, participated in the data collection, analysis, interpretation of the data and drafted the manuscript. RL and DS participated in study design and coordination and helped to draft the manuscript. MSS contributed to the interpretation of the data and revised the manuscript for clarifications. All authors approved the final draft.

## Pre-publication history

The pre-publication history for this paper can be accessed here:

http://www.biomedcentral.com/1471-2296/13/84/prepub
